# Nasal Septum Deviation as the Consequence of BMP-Controlled Changes to Cartilage Properties

**DOI:** 10.3389/fcell.2021.696545

**Published:** 2021-06-24

**Authors:** Pranidhi Baddam, Daniel Young, Garett Dunsmore, Chunpeng Nie, Farah Eaton, Shokrollah Elahi, Juan Jovel, Adetola B. Adesida, Antoine Dufour, Daniel Graf

**Affiliations:** ^1^School of Dentistry, University of Alberta, Edmonton, AB, Canada; ^2^Department of Physiology and Pharmacology, University of Calgary, Calgary, AB, Canada; ^3^Department of Medical Microbiology and Immunology, University of Alberta, Edmonton, AB, Canada; ^4^Department of Medicine, University of Alberta, Edmonton, AB, Canada; ^5^Department of Surgery, University of Alberta, Edmonton, AB, Canada

**Keywords:** nasal septum deviation, hyaline cartilage, elastic cartilage, BMP7, BMP2, Wnt signaling, glucose metabolism, chondrocyte properties

## Abstract

The nasal septum cartilage is a specialized hyaline cartilage important for normal midfacial growth. Abnormal midfacial growth is associated with midfacial hypoplasia and nasal septum deviation (NSD). However, the underlying genetics and associated functional consequences of these two anomalies are poorly understood. We have previously shown that loss of Bone Morphogenetic Protein 7 (BMP7) from neural crest (BMP7^*ncko*^) leads to midfacial hypoplasia and subsequent septum deviation. In this study we elucidate the cellular and molecular abnormalities underlying NSD using comparative gene expression, quantitative proteomics, and immunofluorescence analysis. We show that reduced cartilage growth and septum deviation are associated with acquisition of elastic cartilage markers and share similarities with osteoarthritis (OA) of the knee. The genetic reduction of BMP2 in BMP7^*ncko*^ mice was sufficient to rescue NSD and suppress elastic cartilage markers. To our knowledge this investigation provides the first genetic example of an *in vivo* cartilage fate switch showing that this is controlled by the relative balance of BMP2 and BMP7. Cellular and molecular changes similar between NSD and knee OA suggest a related etiology underlying these cartilage abnormalities.

## Introduction

Nasal septum cartilage that divides the nasal cavity in two is a hyaline cartilage. It resists deformation, provides structural support to the midface, and is the pacemaker for midfacial growth. Nasal septum deviation (NSD) that describes a non-straight, deformed nasal septum is a common abnormality and can be observed in 80% of the population (Serifoglu et al., 2017). NSD might be inconspicuous but frequently is associated with various degrees of nasal airway obstruction resulting in Sleep Disordered Breathing (SDB) (Wang et al., 2016; Alsufyani et al., 2017; Mandour et al., 2019). Congenitally acquired deviation frequently develops during time of rapid midfacial growth and is associated with reduced midfacial growth and a strong predisposition for SDB (D’Ascanio et al., 2010). Another common etiology is trauma to the face and nose. Corrective septoplasties often require graft cartilage, which depending on the site of harvest is either limited in amount, associated with donor site morbidity, or both. Despite the ability to model surgical septoplasty outcomes virtually before surgery (Moghaddam et al., 2020), revision rates remain high and exceed 15% (Lavernia et al., 2019). This high rate may be a consequence of our limited understanding of the etiology of pediatric, congenital acquired NSD and its associated secondary complications (Baddam et al., 2020). It is currently unknown if NSD has an underlying molecular and cellular etiology, even though this might be implied from congenital cases that are associated with reduced midfacial growth.

Although the nasal septum cartilage is a hyaline cartilage, it differs from other hyaline cartilages in several aspects. First, it is an active center for midfacial growth and much of its growth occurs, at least in the mouse, through chondrocyte hypertrophy (Baddam et al., 2021b). Second, hypertrophic chondrocytes situated in the middle part of the septum do not undergo apoptosis or ossification, except in the posterior part to form the bony perpendicular plate of the ethmoid (Wealthall and Herring, 2006). Third, it is a mirrored structure with progenitor cells and immature chondrocytes located on either side of the septum, close to the perichondrium (Baddam et al., 2021b). Fourth, hypertrophic chondrocytes are surrounded by a dense collagen fibril matrix thought to provide rigidity and stiffness. To what degree any of these features are altered in NSD remains unexplored.

Changes to hyaline cartilage in the context of cartilage pathologies are best understood for osteoarthritis (OA) of the knee. There, common accepted features are loss of glycosaminoglycans (GAGs), increase in chondrocyte hypertrophy along with an increase in Collagen X (COL X), Indian Hedgehog (IHH) and Runt-related transcription factor 2 (RUNX2), increase in reactive oxygen species (ROS) production and increase in glucose metabolism (Tchetina and Markova, 2018). Other changes, such as altered elasticity remain controversial, as some studies report a decrease in elasticity while others propose an increase (Coles et al., 2010; Candela et al., 2016). On a molecular level, perturbations to several signaling networks can be observed in knee OA. Reduction of the WNT antagonists Dickkopf1 (DKK1) and Frizzled Related Protein (FRZB) concomitant with an increase in non-phosphorylated Beta-Catenin (NPBC) as readout for canonical WNT signaling have been described (Liu et al., 2016; Zhong et al., 2016b). Alterations to Bone Morphogenetic Protein (BMP) signaling are associated with chondrocyte hypertrophy. BMP7 suppresses while BMP2 promotes chondrocyte hypertrophy and matrix degradation (Caron et al., 2013) and decreased levels of BMP7 have been directly associated with OA (Merrihew et al., 2003; Huang et al., 2018). If and to what degree similar changes occur in cartilage of a deviated septum is not known.

We recently described that mice with neural crest-specific deletion of BMP7 (BMP7^*ncko*^) develop midfacial hypoplasia and nasal airway obstruction (Baddam et al., 2021a). A hallmark of this model is the development of a significantly deviated nasal septum at a juvenile age that is associated with progression to abnormal breathing. Deletion of BMP7 from developing limbs leads to articular cartilage degeneration and synovial inflammation (Abula et al., 2015). Proteoglycan content and aggrecan expression were reduced, while expression of matrix metalloproteinase-13 (MMP13) was increased. In this study, we asked whether similar molecular and cellular changes as observed in the knee are associated with the development of NSD in the BMP7^*ncko*^ mouse.

We demonstrate that BMP7 is expressed in the perichondrium and nasal chondrocytes throughout postnatal development. Loss of BMP7 leads to histomorphological changes by 4 weeks of age, the time the NSD is established. Correlating those changes to molecular changes using gene expression analysis, quantitative shotgun proteomics and immunofluorescence analysis at various developmental time-points, we identified that alterations to chondrocyte properties precede NSD. This included acquisition of elastic cartilage markers, a switch to glucose metabolism, along with increase in molecular markers commonly associated with knee OA. Loss of BMP7 was also associated with a significant increase in canonical WNT signaling in mature chondrocytes. Concomitant reduction of BMP2 in BMP7^*ncko*^ mice restored the change in WNT signaling, prevented the development of the deviation, and rescued the midfacial hypoplasia, demonstrating that the balance of BMP2 and BMP7 synergistically determines cartilage properties. As many of the cellular and molecular changes in NSD in BMP7^*ncko*^ mice share pathophysiological similarities with knee OA, this study sets a precedent for the need to further understand nasal cartilage properties for use in tissue engineering or clinical applications relating to regeneration of damaged cartilage in knee OA.

## Materials and Methods

### Animal Models

Both male and female mice were used for this study. All mice were maintained on the C57BL/6 background and backcrossed for at least 10 generations. *BMP7* expression was identified using BMP7LacZ reporter mice (Malik et al., 2020). BMP7^*fl/fl*^ mice also referred to as BMP7^*ctrl*^ mice, were crossed to WNT1-cre mice to delete BMP7 from neural crest cells (subsequently referred to BMP7^*ncko*^ mice) (Malik et al., 2020). To rescue the NSD, BMP7^*ncko*^ mice were crossed with BMP2^*wt/fl*^ (Malik et al., 2018) to obtain BMP2^*wt/fl*^BMP7^*ncko*^, also referred to as BMP2^*het*^BMP7^*ncko*^ subsequently. Lineage tracing was done using mT/mG mice [*Gt(ROSA)26Sor^TM 4(ACTB–tdTomato,–EGFP)Luo^*/J] (Muzumdar et al., 2007). BMP7 expression and identification of neural crest cells in the nasal septum was conducted at birth (postnatal day 0, P0), 2 weeks (postnatal day 14, P14) and 4 weeks (postnatal day 30, P30). Additionally, nasal septum of BMP7^*ctrl*^ and BMP7^*ncko*^ mice were also assessed at the abovementioned time-points. BMP2^*het*^BMP7^*ncko*^ mice were assessed only at P30.

### Micro-Computed Tomography (μCT) Analysis

Morphological changes to nasal septum of BMP7^*ctrl*^ and BMP7^*ncko*^ (*n* = 3/genotype) were assessed at P14 and P30 using μCT. Acquisition and reconstruction of nasal septum using MILabs μCT at the School of Dentistry, University of Alberta was conducted as previously described (Baddam et al., 2021b). The degree and severity of NSD were quantified on coronal representations of the nasal septum using Amira software. Two landmarks were placed at the abutment where the perpendicular plate meets the cribriform plate of ethmoid bone and where the vomer articulates with the palatine bone, respectively. A straight line was drawn between these two points indicating a hypothetical straight septum, and its length was considered as its height (a). Next, the actual length of the septum was determined by tracing the actual length of the septum (b). The degree of deviation was determined as (b – a)/a and expressed as percentage. Longitudinal experiments to determine whether NSD becomes severe overtime were assessed by calculating the degree of NSD on the same mice twice at two different ages [4 weeks (P30) and 10 weeks (P74)].

### RNA Sequencing

Total RNA from isolated nasal septum of P0 BMP7^*ctrl*^ and BMP7^*ncko*^ mice (*n* = 4/genotype) was extracted with TRIzol reagent (Invitrogen). RNAseq libraries were constructed from 500 ng of total RNA using the NEBNext Ultra II Directional RNA Library Prep Kit for Illumina (NEB). Polyadenylated mRNAs were enriched with oligo dTs conjugated to paramagnetic beads. Enriched mRNAs were fragmented chemically and used for cDNA synthesis. cDNA was end-repaired and A-tailed, ligated to linkers and finally indexed by PCR, to enable multiplexing during sequencing. Sequencing was done on a MiSeq instrument, following a paired-end 75 cycles protocol. Raw sequencing data is publicly available from the NCBI SRA portal under accession number PRJNA622501. The Bioinformatic analysis was conducted where fragments were mapped to the human cDNA database (GRCh38) using Kallisto (Bray et al., 2016), with 100 permutations during pseudo-alignments. Differential expression analysis of RNAseq data was conducted using negative binomial generalized linear models with the edgeR R package (Love et al., 2014). Gene abundance differences with a corrected *p*-value < 0.05 and a log fold change of ≤–2 or ≤2 were considered differentially expressed. Mean-differential plots were generated with edgeR and heatmaps were generated using the R package pheatmap. Gene ontologies were identified using PANTHER gene ontology consortium (Mi et al., 2019). Only significant ontologies were selected for representation (FDR < 0.05).

### Shotgun Proteomics

Nasal septum of P14 and P30 BMP7^*ctrl*^ and BMP7^*ncko*^ mice (*n* = 4/genotype) were isolated and frozen until processing for proteomics experiments. Protein lysates were obtained by tissue lysis in buffer containing 1% SDS, in 200 mM HEPES (pH 8.0) and cOmplete^TM^ Protease Inhibitor Cocktail (MilliporeSigma, Oakville, ON, Canada). Samples were reduced with 10 mM dithiothreitol (DTT) for 30 min at 37°C. Once cooled at room temperature (RT), cysteine alkylation was achieved by incubation with a final concentration of 15 mM iodoacetamide for 25 min in the dark at RT. Next, samples were precipitated in acetone/methanol, washed three times in methanol, and trypsinized (Trypsin Gold from Promega, Madison, WI, United States). The pH was adjusted to 6.0 with HCl. To label peptide α- and ε-amines, samples were incubated for 18 h at 37°C with isotopically heavy [40 mM 13CD2O + 20 mM NaBH3CN (sodium cyanoborohydride)] or light labels [40 mM light formaldehyde (CH2O) + 20 mM NaBH3CN]. Next, samples were combined and subjected to C18 chromatography before being run on liquid chromatography and tandem mass spectrometry.

#### High-Performance Liquid Chromatography (HPLC) and Mass Spectrometry (MS)

The liquid chromatography and mass spectrometry experiments were carried out by the Southern Alberta Mass Spectrometry (SAMS) core facility at the University of Calgary, Canada. The analysis was performed on an Orbitrap Fusion Lumos Tribrid mass spectrometer (Thermo Scientific) operated with Xcalibur (version 4.0.21.10) and coupled to a Thermo Scientific Easy-nLC (nanoflow liquid chromatography) 1200 system. Tryptic peptides (2 μg) were loaded onto a C18 column (75 μm × 2 cm; Acclaim PepMap 100, P/N 164946; Thermo Scientific) at a flow rate of 2 μL/min of solvent A (0.1% formic acid and 3% acetonitrile in LC-MS grade water). Peptides were then electrosprayed using 2.3 kV voltage into the ion transfer tube (300°C) of the Orbitrap Lumos operating in the positive mode. The Orbitrap first performed a full MS scan at a resolution of 120,000 fwhm to detect the precursor ion having a m/z between 375 and 1,575 and a +2 to +7 charge. The Orbitrap AGC (Auto Gain Control) and the maximum injection time were set at 4 × 10^5^ and 50 ms, respectively. The Orbitrap was operated using the top speed mode with a 3 s cycle time for precursor selection. The most intense precursor ions presenting a peptidic isotopic profile and having an intensity threshold of at least 5,000 were isolated using the quadrupole and fragmented with HCD (30% collision energy) in the ion routing multipole. The fragment ions (MS2) were analyzed in the ion trap at a rapid scan rate. The AGC and the maximum injection time were set at 1 × 10^4^ and 35 ms, respectively, for the ion trap. Dynamic exclusion was enabled for 45 s to avoid the acquisition of the same precursor ion having a similar m/z (±10 ppm).

#### Proteomic Data and Bioinformatics Analysis

Spectral data were matched to peptide sequences in the murine UniProt protein database using the Andromeda algorithm (Cox et al., 2011) as implemented in the MaxQuant software (Cox and Mann, 2008) package v.1.6.0.1, at a peptide-spectrum match false discovery rate (FDR) of <0.01. Search parameters included a mass tolerance of 20 ppm for the parent ion, 0.5 Da for the fragment ion, carbamidomethylation of cysteine residues (+57.021464 Da), variable N-terminal modification by acetylation (+42.010565 Da), and variable methionine oxidation (+15.994915 Da). N-terminal and lysine heavy (+34.063116 Da) and light (+28.031300 Da) dimethylation were defined as labels for relative quantification. The cleavage site specificity was set to Trypsin/P, with up to two missed cleavages allowed. Significant outlier cutoff values were determined after log(2) transformation by box-and-whisker analysis using the BoxPlotR tool (Altman and Krzywinski, 2016). A minimum of two distinct peptides per protein for the quantification was used. Following an interquartile boxplot analysis, proteins with a quantification ratio of under 0.65 and above 1.5 were considered for the analysis. The proteomic data has been deposited to the ProteomeXchange Consortium via the PRIDE partner repository with the data set identifier px-submission PXD024813. A list of all differentially regulated proteins is provided in the Supplementary Tables 1, 2.

### RNA Extraction Quantitative Real-Time Polymerase Chain Reaction (qRT-PCR)

Nasal septum from P0, P14 and P30 BMP7^*ctrl*^ and BMP7^*ncko*^ mice (*n* = 3/genotype) were dissected and processed for RNA extraction. RNA extraction and qRT-PCR were conducted as previously described (Malik et al., 2020). Fold difference was determined in relation to the housekeeping gene 36B4 using ΔΔCt method (Malik et al., 2020). Analysis was conducted on biological and technical triplicates. Data shown are representatives from three biological samples per genotype. A list of primer pairs used for gene expression analysis is outlined in Supplementary Table 3.

### Tissue Processing and Histology

BMP7^*ctrl*^ and BMP7^*ncko*^ mice (*n* = 3/genotype/age) were fixed using 4% paraformaldehyde (PFA). Mice skulls were dissected and decalcified in 0.5M ethylenediaminetetraacetic acid (EDTA) as previously described (Malik et al., 2020). Mouse skulls were frontally embedded in paraffin and sectioned using 820 Spencer microtome at 7 microns. For histological staining, sections were deparaffinized as previously described (Baddam et al., 2021b).

#### Histological Stains

Medial nasal septum sections were used for histological and immunofluorescence analysis (Baddam et al., 2021b). Hematoxylin and Eosin (H&E) and Safranin O staining on nasal septum of P14 and P30 BMP7^*ctrl*^ and BMP7^*ncko*^ mice (*n* = 3/genotype/age) were performed as previously described (Malik et al., 2020; Baddam et al., 2021b). Picrosirius staining was performed using Sirius Red staining protocol from IHC world (Kiernan, 2001). H&E was performed to assess gross morphology. Safranin O staining was used to stain cartilage and Picrosirius Red staining was used to stain collagen fibers. Images were acquired on an Olympus IX73 microscope using 20X and 40X objectives.

#### Immunofluorescence

Immunofluorescent staining was performed on medial nasal septum paraffin sections of P0, P14, P30 BMP7^*ctrl*^ and BMP7^*ncko*^ and P30 BMP2^*het*^BMP7^*ncko*^ mice as previously described (Baddam et al., 2021b). Specifications of primary and secondary antibodies are provided in Supplementary Table 4. Images were acquired on an Olympus IX73 microscope using 20x objectives. Analysis was performed on three biological replicates. Negative controls demonstrating background staining were previously demonstrated (Baddam et al., 2021b).

### LacZ Staining

P0, P14, and P30 BMP7LacZ reporter mice (*n* = 3/genotype/age) were stained using previously described staining procedure (Malik et al., 2020). Mouse skulls were fixed, decalcified, processed as described above. The paraffin sections were counterstained using Safranin O staining protocol.

### Statistics

Graphs indicate individual measurements as well as mean ± standard deviation when applicable. A two-tailed independent *t*-test was conducted to test for statistical significance between BMP7^*ctrl*^ and BMP7^*ncko*^ mice. Microsoft Excel was used to display graphs and an independent unpaired *t*-test was conducted using online statistical software (Socscistatistics, 2020). ^∗^*P* < 0.05; ^∗∗^*P* < 0.01; ^∗∗∗^*P* < 0.001; ns, not significant *P* > 0.05.

### Study Approval

This study was approved by the Health Sciences Animal Care and Use Committee at the University of Alberta (protocol #: AUP1149). All experiments in this study were conducted in accordance with the Canadian Council on Animal Care guidelines.

## Results

### BMP7 Is Expressed in Nasal Cartilage and Its Deletion Results in Nasal Septum Deviation

**FIGURE 1:**
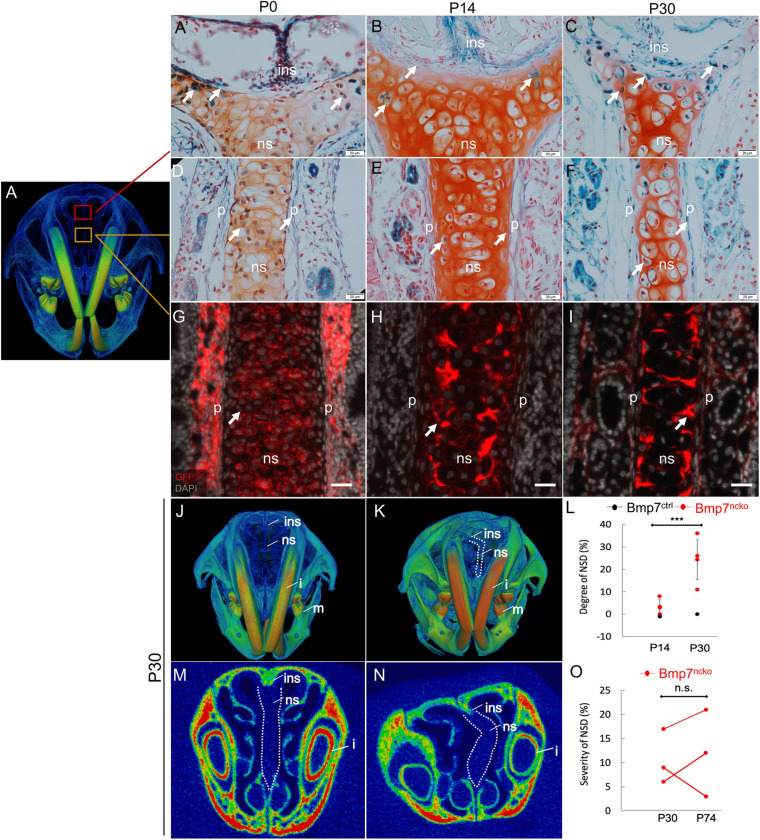
Deletion of BMP7 in neural crest cells results in nasal septum deviation (NSD). **(A)** μCT reconstruction of P30 mouse skull demonstrating the location of nasal septum where images were acquired from. LacZ stained (blue) nasal septum paraffin sections demonstrating BMP7 expression in P0 **(A’,D)**, P14 **(B,E)**, and P30 **(C,F)** Bmp7LacZ reporter mice. Neural crest cells in the nasal septum of P0 **(G)**, P14 **(H)**, and P30 **(I)** identified by Green Fluorescent Protein (GFP) (red) antibody. DAPI used to stain nuclei (gray). Scale bar = 50 μm. 3D frontal view of P30 Bmp7^*ctrl*^ **(J)** and Bmp7^*ncko*^ **(K)** mice demonstrating nasal septum deviation as outlined in white. **(L)** Quantification of NSD in P14 and P30 mice (*n* = 4/genotype/age). 2D frontal cross section of nasal septum in Bmp7^*ctrl*^ **(M)** and Bmp7^*ncko*^ **(N)** mice. **(O)** Quantification of nasal septum deviation in Bmp7^*ncko*^ mice over time (*n* = 3/age). Data points represent individual mice with error bars demonstrating biological variation. ins, internasal suture; ns, nasal septum; p, perichondrium; i, incisor; m, mandible. P14, postnatal day 14; P30, postnatal day 30; P74, postnatal day 74. ***p < 0.001, two-tailed independent *t*-test. n.s., not significant. Scale bar **(A–F)**: 20 μm and **(G,H)**: 50 μm.

Anterior and medial nasal septum sections were used for histological assessment of the nasal septum (Baddam et al., 2021b). Images were acquired at the abutment of nasal septum with internasal suture as well as the middle of the nasal septum (Figure 1A). To identify when and where BMP7 is expressed in the nasal septum, we used BMP7LacZ reporter mice (*n* = 3/age). Expression was observed at the abutment at all ages investigated [Figures 1A’–C; Post-natal day 0 (P0), Post-natal day 14 (P14), Post-natal day 30 (P30)]. In the middle region of the septum, BMP7 was clearly expressed in chondrocytes as well as the perichondral lining from P0 onward (Figures 1D–F). The expression was variable over time, with least expression observed in chondrocytes at P14 (Figure 1E). Lineage tracing confirmed neural crest origin of septum chondrocytes and perichondrium (*n* = 3/age) (Figures 1G–I). As previously shown (Baddam et al., 2021a), the nasal septum in BMP7^*ncko*^ mice becomes severely deviated by P30 (Figures 1J–O) (*n* = 3/age/genotype) with the degree of deviation reaching between 10 and 35% (Figure 1L; *p* < 0.001) and variable in severity over time (Figure 1O). The nasal septum in the mouse grows rapidly between P7–P14 and P21–P30 but shows a significant reduction between P14 and P21 (Vora et al., 2015; Baddam et al., 2021b). As BMP7^*ncko*^ mice develop midfacial hypoplasia in addition to NSD, we suspected that changes to cartilage appearance and properties are associated with the development of these two pathologies.

### Apoptosis Precedes Nasal Septum Deviation and Reduced GAGs Observed in the Deviated Septum

**FIGURE 2:**
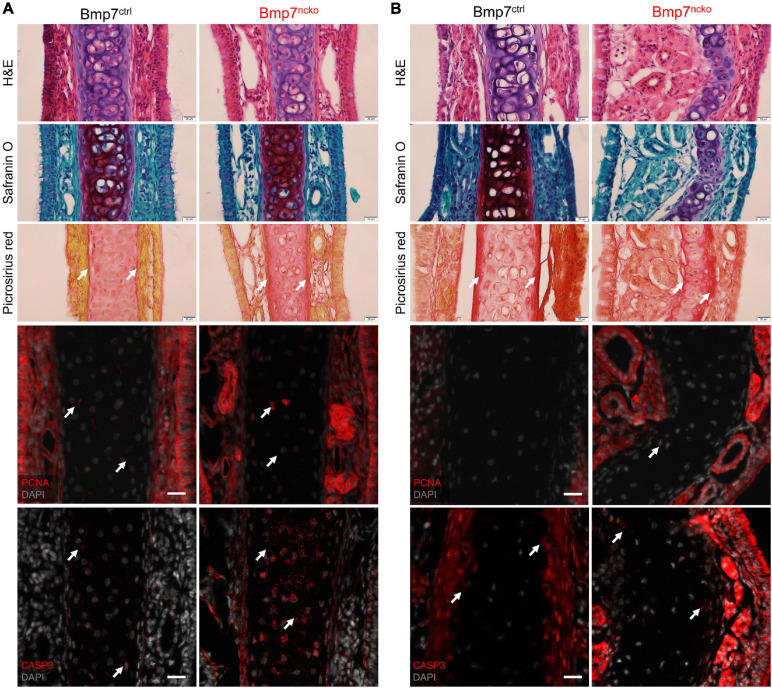
Cell death altered glycosaminoglycan and collagen fibril organization observed in Bmp7^*ncko*^ mice. Histological staining on nasal septum paraffin sections at P14 **(A)** and P30 **(B)** timepoints (*n* = 3/genotype/age). Hematoxylin (purple) and Eosin (pink) (H&E) staining for gross morphology of the nasal septum. Representative Safranin O (red) staining for cartilage and Picrosirius Red (pinkish-red) staining for collagen fibers. Reduction in Safranin O staining was observed only in P30 Bmp7^*ncko*^ mice. To identify proliferation in the nasal septum, immunofluorescence staining using PCNA antibody (red) was performed with sections counterstained with DAPI for nuclei (gray). No changes to proliferation were observed. Apoptosis was characterized using CASP3 antibody (red) and counterstained with DAPI for nuclei (gray). Apoptosis precedes nasal septum deviation in Bmp7^*ncko*^ mice. PCNA, proliferating cell nuclear antigen; CASP3, cleaved caspase 3. Arrows denoting positive stain/signal. Scale bar (histological stains) = 20 μm and (Immunos) = 50 μm.

At P14, histological analysis of the nasal septum using H&E, Safranin O and Picrosirius Red showed a comparable cellular organization with round (mature/hypertrophic) cells restricted to the middle and flat (immature/chondrocyte progenitors) cells located at the periphery (Figure 2A; *n* = 3/genotype). At P30, BMP7^*ncko*^ cartilage had lost this clear organization. The degree of hypertrophy was reduced, fewer flat cells were evident at the periphery. Safranin O staining was reduced indicative of a reduction in GAGs. Deposition of collagen fibers was asymmetric with increased deposition on the outer side of the deviation and a reduction on the contralateral side (Figure 2B, *n* = 3/genotype). Cellular proliferation (PCNA) was unaffected. Apoptosis, measured by cleaved Caspase 3 staining, was increased at P14 only with no apparent differences at P30. Thus, a burst of cell apoptosis precedes cellular disorganization and reduction in GAGs, indicating that changes to chondrocyte properties might predispose to the development of NSD.

### Proteins Involved in Extracellular Matrix Organization and Cell Metabolism Deregulated Before Nasal Septum Deviation

**FIGURE 3:**
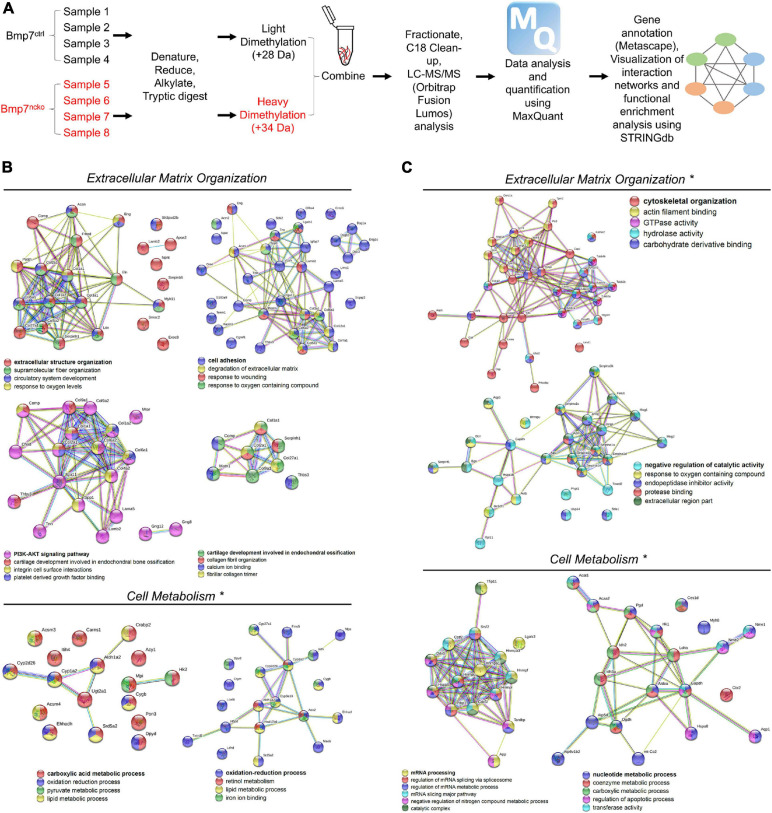
Altered extracellular matrix (ECM) organization and cell metabolism precede nasal septum deviation in Bmp7^*ncko*^ mice. **(A)** Workflow of Proteomics experiment and analysis (*n* = 4/age/genotype). STRINGdb software was used to identify protein-protein interactions. **(B)** Differentially expressed proteins and their associated biological processes altered in P14 Bmp7^*ncko*^ mice in comparison to Bmp7^*ctrl*^. **(C)** Differentially expressed proteins and their associated biological processes altered in P30 Bmp7^*ncko*^ mice in comparison to Bmp7^*ctrl*^. * Denotes proteins upregulated in Bmp7^*ncko*^ mice. Proteins involved in retinoic acid signaling and lipid metabolism were upregulated at 2 weeks whereas proteins involved in glucose metabolism were upregulated at 4 weeks. A list of all differentially regulated proteins is provided in the Supplementary Tables 1, 2.

We performed shotgun proteomics (Figure 3A) on isolated P14 and P30 BMP7^*ctrl*^ and BMP7^*ncko*^ nasal septum (*n* = 4/genotype/age) to identify potential proteome changes in the mutant septum before and following septum deviation. STRING database^1^ was used to assign ontology and map protein-protein interactions. At P14, several clusters reflecting a decrease in proteins involved in extracellular structure organization, cell adhesion, and degradation of extracellular matrix were identified (Figure 3B), exemplified by Elastin (ELN), Collagen I (COL 1), Collagen II (COL 2), Osteopontin (SPP1), and Aggrecan (ACAN). Several of those proteins as well as reduced mTOR associate with the PI3K-AKT signaling pathway, indicating a reduction of this important pathway regulating cell proliferation, survival, and metabolism (Yu and Cui, 2016). At the same time, a cluster of proteins involved in retinoic acid signaling and lipid metabolism, such as CYP1A2, and ALDH1A2, was upregulated. Together this suggested that loss of BMP7 is associated with a change in cell metabolism at P14.

At P30, when NSD is established, a rather different picture of proteome changes was observed (Figure 3C). An increase in proteins involved in the response to oxygen-containing compounds, catalytic activity, and cytoskeletal organization such as Decorin (DCN) was observed in BMP7^*ncko*^ mice. In addition, changes in mRNA processing and nucleotide metabolic processes were observed. Of interest, the increase in Hexokinase 1 (HK1), Aldolase A (ALDOA) and Glyceraldehyde 3-phosphate dehydrogenase (GAPDH) indicated an increase in glycolysis and proteins involved in RNA metabolism, such as Heterogeneous nuclear ribonucleoprotein U (HNRPU), were also upregulated. In summary, we identified global dynamic changes to several cellular processes in BMP7-mutant nasal cartilage, most prominently extracellular matrix and cell metabolism both prior to and once NSD is established. However, spatial information on the expression of these proteins in the septum is not known.

### Increase in Chondrocyte Hypertrophy and Acquisition of Elastic Cartilage Markers in Deviated Nasal Septum of BMP7^*ncko*^ Mice

**FIGURE 4:**
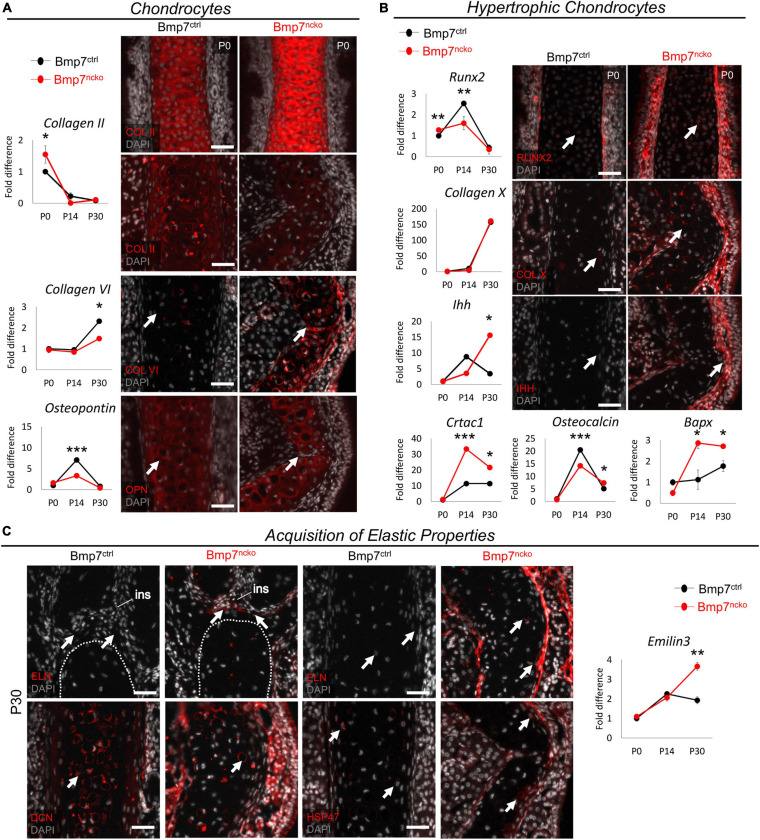
Bmp7^*ncko*^ mice undergo chondrocyte hypertrophy and acquire elastic cartilage markers. **(A)** qRT-PCR of dissected nasal septum demonstrating differential gene expression of Collagen II (COL II), Collagen VI (COL VI), and Osteopontin (OPN) at P0, P14, and P30 timepoints. Immunofluorescence staining of COL II revealed an increase at P0 (top panel) and a decrease at P30 (bottom panel). Expression of COL VI was increased in the Bmp7^*ncko*^ mice while reduced expression of OPN was observed in P30 mutant mice via immunofluorescence. **(B)** Differential gene expression of genes involved in chondrocyte hypertrophy RUNX2, Collagen X (COL X), Indian hedgehog (IHH), Cartilage acidic protein 1 (CRTAC1), Osteocalcin, and Bapx indicating Bmp7^*ncko*^ mice undergo chondrocyte hypertrophy. Immunofluorescence staining of P0 nasal septum demonstrates an increase in RUNX2 expression in addition to an increase in expression of COL X and IHH in P30 Bmp7^*ncko*^ mice. **(C)** Expression of Elastin (ELN) was observed in the abutment of the nasal septum and internasal suture (ins) as well as in the middle of the nasal septum in P30 Bmp7^*ncko*^ mice. EMILIN3 expression was also significantly increased at P30 in the mutant mice. Expression of Decorin (DCN) and SERPINH1 (HSP47) were also identified to be increased in the mutant nasal septum. Independent two-tailed *t*-test was performed with significance denoted as *: *p* < 0.05, **: *p* < 0.01, ***: *p* < 0.001. (*n* = 3/genotype/age). Antibody expression demonstrated in red and DAPI stained nuclei in gray for all immunofluorescence images. Arrows denoting positive antibody signal. Scale bar = 50 μm.

Gene expression analysis and immunofluorescence staining (*n* = 3/age/genotype) was performed using antibodies against proteins identified in the proteomics data. We assessed expression of extracellular matrix components associated with hyaline cartilage, cartilage hypertrophy, and OA at P0, P14, P30 to capture the timing of any changes. We additionally compared these protein changes to changes in gene expression (Figure 4A). COL II was significantly increased at P0 BMP7^*ncko*^ mice (*p* < 0.05) but became comparable by P30. Expression of COL VI, a pericellular matrix gene (Zelenski et al., 2015), was initially comparable but showed a significant reduction at P30 (*p* < 0.05). However, protein expression was increased at the site of deviation. OPN, a gene involved in early chondrogenesis (Gerstenfeld and Shapiro, 1996; Baddam et al., 2021b), was significantly reduced in P14 mutant nasal septum. Additionally, at P30, OPN protein expression was lost in a subset of cells that appear to extend from the perichondrium at the site of deviation. Thus, loss of BMP7 alters expression of several extracellular matrix components associated with chondrogenesis.

RUNX2 (Caron et al., 2013), COL X, IHH, CRTAC1 (Sanchez et al., 2017), Osteocalcin, and BAPX (Caron et al., 2015) have all been associated with hypertrophic differentiation of chondrocytes, with RUNX2, COL X, and IHH being the most commonly assessed proteins for chondrocyte hypertrophy. Although RUNX2 gene expression (Figure 4B) was slightly increased at P0 (*p* < 0.01), it did not show the increase seen in control mice at P14 (*p* < 0.01). BMP7^*ncko*^ mice indicate early induction of chondrocyte hypertrophy. Antibody staining for RUNX2 at P0 confirmed increased expression. While expression of COL X was comparable at all stages, expression of IHH was significantly increased (*p* < 0.05) in the mutant septum at P30. Antibody staining showed increased COL X and IHH in the deviated septum. Expression of both CRTAC1, a gene involved in chondrocyte differentiation (Sanchez et al., 2017), and BAPX, an inhibitor of chondrocyte hypertrophy (Caron et al., 2015), was significantly upregulated at P14 and P30 (^∗^*p* < 0.05, ^∗∗∗^*p* < 0.001). Osteocalcin previously described to accumulate at the onset of hypertrophy (Lian et al., 1993) was significantly decreased at P14 (*p* < 0.001) but increased at P30 (*p* < 0.05) in BMP7^*ncko*^ mice. These observations indicate that the development of NSD appears to be associated with altered regulation of chondrocyte hypertrophy.

A key function of hyaline cartilage is to provide stiffness and rigidity to the nasal cavity. Given the significant molecular changes that precede the development of the deviation, we wondered whether the structural deformation might be associated with the acquisition of elastic cartilage markers that are not normally seen in hyaline cartilage. Immunofluorescence staining for Elastin (ELN), a protein abundant in elastic cartilage, revealed that loss of BMP7 resulted in Elastin expression at the abutment and the middle region of the septum, suggesting that at least parts of the nasal septum indeed might have acquired elastic cartilage properties (Figure 4C). This potential switch to elastic cartilage markers in P30 BMP7^*ncko*^ mice was supported by an increase in Emilin3 expression (*p* < 0.01), another characteristic elastic cartilage gene (Schiavinato et al., 2012). Additional evidence for structural changes and alterations to collagen fibrils (Figure 2B) came from the altered distribution of Decorin (DCN) and HSP47 (Ishikawa et al., 2018) in P30 BMP7^*ncko*^ mice.

Collectively, our data suggest that NSD is associated with altered cartilage properties, in particular a gain in expression of elastic cartilage markers.

### Increase in Glycolytic Activity as a Consequence of Altered WNT and BMP Signaling

**FIGURE 5:**
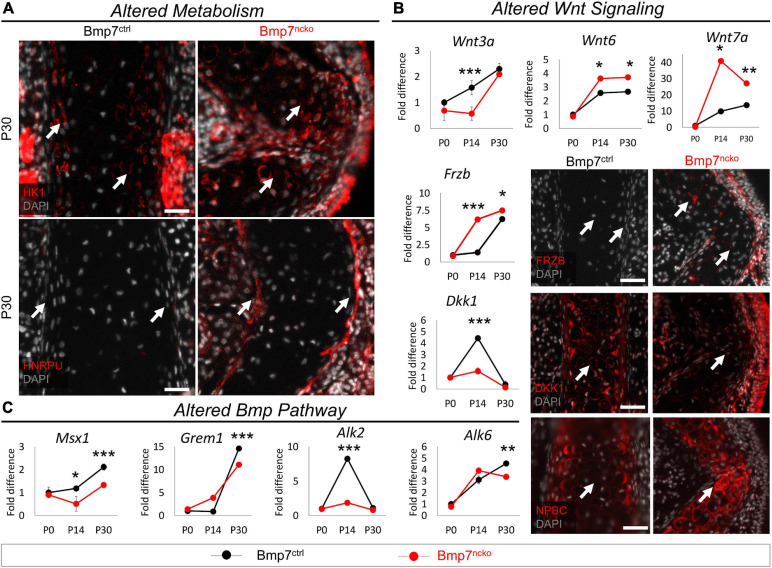
Altered metabolism and Wnt signaling in P30 Bmp7^*ncko*^ mice. **(A)** Immunofluorescence staining of Hexokinase (HK1) and Heterogeneous nuclear ribonucleoprotein U (HNRPU) in P30 Bmp7^*ctrl*^ and Bmp7^*ncko*^ mice revealed an increase in expression of both proteins in the mutant mice. **(B)** qRT-PCR of dissected nasal septum at P0, P14 and P30 timepoints demonstrating differential gene expression of Wnt ligands (WNT3A, WNT6, WNT7A) and Wnt antagonists (FRZB, DKK1). Immunostaining of Wnt antagonists (FRZB, DKK1) in P30 Bmp7^*ctrl*^ and Bmp7^*ncko*^ mice demonstrate an increase in expression of FRZB and decrease in DKK1 expression in the mutant mice. Immunofluorescence staining using Non-phosphorylated Beta-Catenin (NPBC) in P30 mice demonstrates nuclear expression in Bmp7^*ncko*^ and cytoplasmic expression in Bmp7^*ctrl*^ mice. **(C)** qRT-PCR of nasal septum at P0, P14, and P30 ages revealed alterations to genes in the Bmp pathway (MSX1, GREM1, ALK2, ALK6). Independent two-tailed *t*-test was performed with significance denoted as *: *p* < 0.05, **: *p* < 0.01, ***: *p* < 0.001. (*n* = 3/genotype/age). Antibody expression demonstrated in red and DAPI stained nuclei in gray for all immunofluorescence images. Arrows denoting positive antibody signal. Scale bar = 50 μm.

Proteome analysis at P30 revealed an increase in several key enzymes in the glycolysis pathway [(HK1, ALDOA, GAPDH, and Lactate Dehydrogenase B (LDHB)] (Lee et al., 2018) in the mutant septum (Figure 3C). Immunofluorescence analysis of HK1 confirmed the increased expression in the deviated nasal septum (Figure 5A). Proteins involved in RNA metabolism like HNRPU were also identified (Figure 3C), and its increased expression was confirmed in P30 BMP7^*ncko*^ mice. The glycolysis pathway is under control of WNT and BMP signaling pathways (Lee et al., 2018). Probing for changes in these two pathways, we identified altered expression of various WNT ligands (WNT3A, WNT6, WNT7A), WNT antagonists (FRZB, DKK1) culminating in increased WNT signaling (NPBC) in mature chondrocytes of the P30 mutant septum (Figure 5B). In general, the changes were largest at P14. WNT3A involved in canonical WNT signaling was decreased at P14 (*p* < 0.001). The two other canonical WNT ligands WNT6 and WNT7A were upregulated at P14 (*p* < 0.05) and P30 (*p* < 0.01) (Huang et al., 2018). The WNT-antagonists FRZB and DKK1 were upregulated and downregulated, respectively, in P14 mutant mice (*p* < 0.001) and to a lesser degree at P30 (*p* < 0.05). Overall, canonical WNT signaling apparent by nuclear NPBC staining was increased in the mutant septum, particularly at the site of deviation, whereas cytoplasmic NPBC staining was observed in BMP7^*ctrl*^ mice. Loss of BMP7 was associated with a reduction in MSX1, a gene downstream of BMP signaling that promotes chondrogenic differentiation of NCC (Méndez-Maldonado et al., 2018), at both P14 (*p* < 0.05) and P30 (*p* < 0.001) timepoints (Figure 5C). Gremlin 1 (GREM1), a BMP antagonist and inhibitor of chondrocyte hypertrophy (Zhong et al., 2016a), was significantly decreased once septum deviation was established. The BMP receptors ALK2 and ALK6, previously described to regulate chondrogenic differentiation (Caron et al., 2013), were decreased at P14 (ALK2; *p* < 0.001) and P30 (ALK6; *p* < 0.01), respectively.

Thus, the development of NSD in BMP7^*ncko*^ mice is accompanied by a switch toward glycolysis along with dynamic changes in WNT and BMP signaling pathways.

### Loss of BMP7 Affects Cell Differentiation and Cell Metabolism Already in P0 Nasal Septum

**FIGURE 6:**
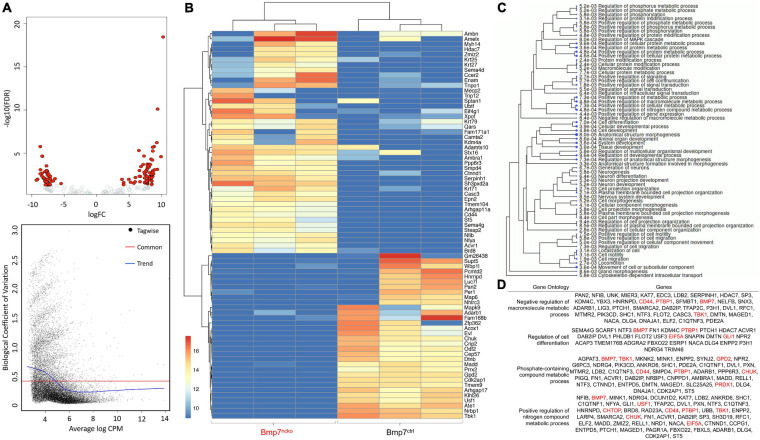
RNA Sequencing analysis of P0 Bmp7^*ctrl*^ and Bmp7^*ncko*^ mice demonstrating differentially expressed genes (*n* = 3/genotype). **(A)** Volcano plot demonstrating genes differentially upregulated and downregulated. Additionally, the biological coefficient of variation plot indicating biological variation within the samples to be between 0.4 and 0.5. **(B)** Heat-map outlining differentially expressed genes between Bmp7^*ctrl*^ and Bmp7^*ncko*^ mice. **(C)** Gene ontology (GO) terms of genes identified to be differentially expressed. **(D)** Specific GO terms and their associated genes. Altered metabolic was a prominent GO term identified in the RNASeq analysis. Genes in red have been validated using qRT-PCR (Figure 7).

Above experiments revealed that the most significant changes to gene expression could be observed at P14. However, some changes were already evident at birth. To systematically identify gene expression changes at P0, RNA-sequencing was performed on dissected P0 BMP7^*ctrl*^ and BMP7^*ncko*^ nasal septa (*n* = 3/genotype). A Volcano plot identified both upregulated and downregulated genes, while the biological coefficient of variation within samples was between 0.4 and 0.5, within an acceptable range variation (Figure 6A). A heat map following unsupervised, hierarchical clustering was created (Figure 6B), and gene ontology (GO) was used to map differentially expressed genes to cellular processes, such as cell metabolism, cell differentiation, and cell and structure morphogenesis (Figure 6C). Validation of differentially expressed genes identified from RNA-Seq analysis demonstrated alterations to proliferation, apoptosis, cell metabolism, production of ROS, signs of chondrocyte hypertrophy and BMP signaling. Individual genes validated by RT-qPCR mapped to significantly altered gene ontologies are shown in Figure 6D.

### Early Signs of Altered Cell Proliferation, Apoptosis, Metabolism and Chondrocyte Hypertrophy in BMP7^*ncko*^ Mice

**FIGURE 7:**
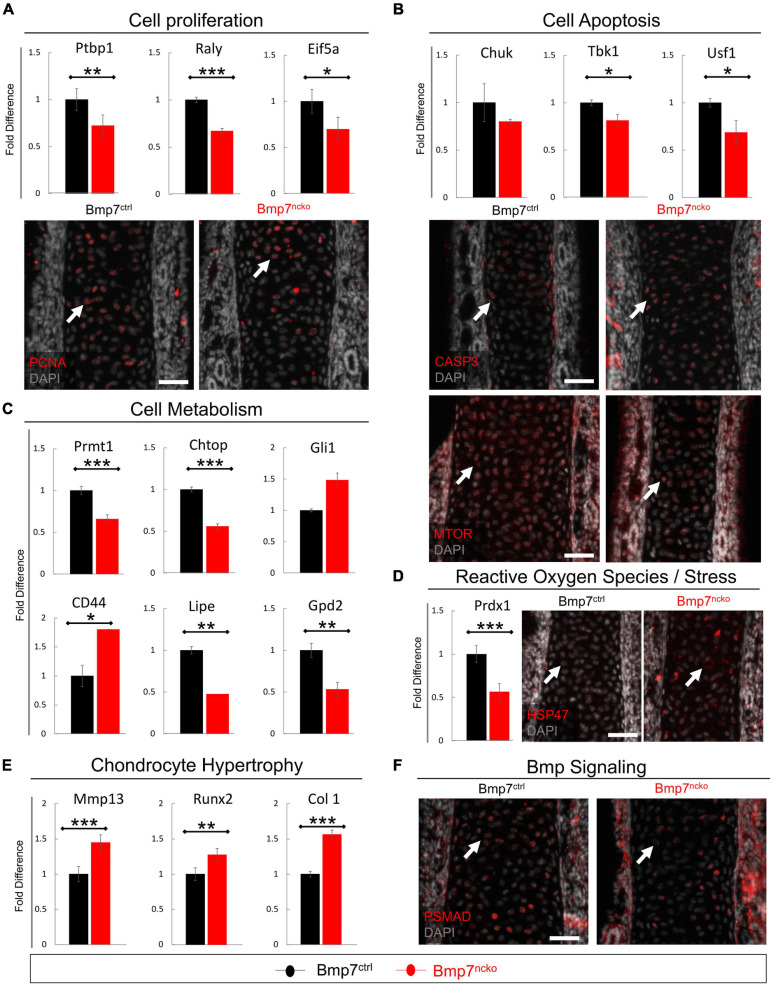
Altered cell proliferation, apoptosis and Bmp signaling precede nasal septum deviation. qRT-PCR validation on P0 dissected nasal septum of genes identified in RNASeq analysis and immunofluorescence staining revealed alteration to **(A)** cell proliferation as evident by reduced PTBP1, RALY, and EIF5a as well as PCNA expression in Bmp7^*ncko*^ mice. **(B)** Increased cell apoptosis was observed as demonstrated by reduced expression of CHUK, TBK1, USF1, increased CASP3 expression and reduced mTOR expression. **(C)** Alterations to lipid metabolism were observed by a significant reduction in PRMT1, CHTOP, LIPE, and GPD2 with an increase in CD44 expression. **(D)** A reduction in expression of PRDX1 and an increase in expression of stress protein HSP47 was observed. **(E)** An increase in MMP13, RUNX2, and COL 1 was observed in Bmp7^*ncko*^ mice. **(F)** Bmp signaling assessed using immunofluorescence staining of PSMAD revealed a decrease in pSMAD in P0 Bmp7^*ncko*^ mice. PTBP1, Polypyrimidine tract-binding protein 1; RALY, Heterogeneous Nuclear Ribonucleoprotein; EIF5A, Eukaryotic Translation Initiation Factor 5A; CHUK, Component Of Inhibitor Of Nuclear Factor Kappa B Kinase Complex; TBK1, TANK Binding Kinase 1; USF1, Upstream Transcription Factor 1; PRMT1, Protein Arginine Methyltransferase 1; CHTOP, Chromatin Target Of PRMT1; GLI1, GLI Family Zinc Finger 1; LIPE, Lipase E; GPD2, Glycerol-3-Phosphate Dehydrogenase 2; PRDX1, Peroxiredoxin 1; MMP13, matrix metalloproteinase 13; RUNX2, RUNX Family Transcription Factor 2; COL 1, Collagen I; PCNA, Proliferating Cell Nuclear Antigen; CASP3, Cleaved Caspase 3; MTOR, Mammalian Target of Rapamycin; HSP47, SERPINH1; pSMAD, Phosphorylated Smad. Independent two-tailed *t*-test was performed with significance denoted as *: *p* < 0.05, **: *p* < 0.01, ***: *p* < 0.001. (*n* = 3/genotype/age). Antibody expression demonstrated in red and DAPI stained nuclei in gray for all immunofluorescence images. Arrows denoting positive antibody signal. Scale bar = 50 μm.

We first confirmed changes to proliferation and apoptosis in P0 septa from BMP7^*ctrl*^ and BMP7^*ncko*^ mice (*n* = 3/genotype). Heterogeneous Nuclear Ribonucleoprotein I (PTBP1) (Cheung et al., 2009), RALY Heterogeneous Nuclear Ribonucleoprotein (RALY) (Cornella et al., 2017) and Eukaryotic Translation Initiation Factor 5A (EIF5A) (Nishimura et al., 2005) known to promote cell proliferation were all significantly reduced in BMP7^*ncko*^ mice (*p* < 0.01, *p* < 0.001, *p* < 0.05) (Figure 7A). Staining for PCNA confirmed the reduction in chondrocyte proliferation. In parallel, expression of genes promoting cell survival, such as Component Of Inhibitor Of Nuclear Factor Kappa B Kinase (CHUK) (Culley et al., 2019), TANK Binding Kinase 1 (TBK1) (Xu et al., 2018) and Upstream Transcription Factor 1 (USF1) (Guo et al., 2018), was also significantly reduced (*p* < 0.05) except for CHUK (Figure 7B). This was mirrored by increased levels of cleaved Caspase-3 (CASP3) and a reduction in mTOR (Guo et al., 2018).

Protein Arginine Methyltransferase 1 (PRMT1), Chromatin Target Of PRMT1 (CHTOP) and GLI Family Zinc Finger 1 (GLI1) impact glucose metabolism (Iwasaki and Yada, 2007; vanLieshout and Ljubicic, 2019). Both PRMT1 and CHTOP were significantly reduced (*p* < 0.001) suggestive of impaired glucose metabolism already at P0. Conversely, genes involved in lipid metabolism and production of triglycerides and cholesterol (CD44 (Jiang et al., 2020), Lipase E (LIPE) (Gu et al., 2019), mitochondrial Glycerol 3 Phosphate Dehydrogenase (GPD2) (Mráček et al., 2013) were also altered. While CD44 was significantly increased (*p* < 0.001), LIPE and GPD2 were significantly reduced (*p* < 0.01) in the mutant septa (Figure 7C). Altered cell metabolism in chondrocytes has been associated with the production of ROS and stress response (Gibson et al., 2008). Indeed, Peroxiredoxin-1 (PRDX1), a ROS scavenger (Kumar et al., 2018), was significantly reduced (*p* < 0.001). Heat shock protein 47 (HSP47), an endoplasmic collagen-binding stress protein, was increased in BMP7^*ncko*^ septa (Figure 7D). ROS can induce chondrocyte hypertrophy (Morita et al., 2007) in a BMP-dependant manner (Kumar et al., 2018). Several indicators for chondrocyte hypertrophy were indeed increased [Matrix metalloproteinase-13 (MMP13) (*p* < 0.001), RUNX2 (*p* < 0.01), Collagen I (COL 1) (*p* < 0.001)] (Figure 7E), while phosphorylated SMAD1/5/8 (pSMAD1/5/8) was overall reduced (Figure 7F). Collectively, this establishes that loss of BMP7 changes the developmental trajectory of nasal chondrocytes already at birth long before septum deviation is manifested.

### Reduction of BMP2 in BMP7^*ncko*^ Mice Rescues Nasal Septum Deviation

**FIGURE 8:**
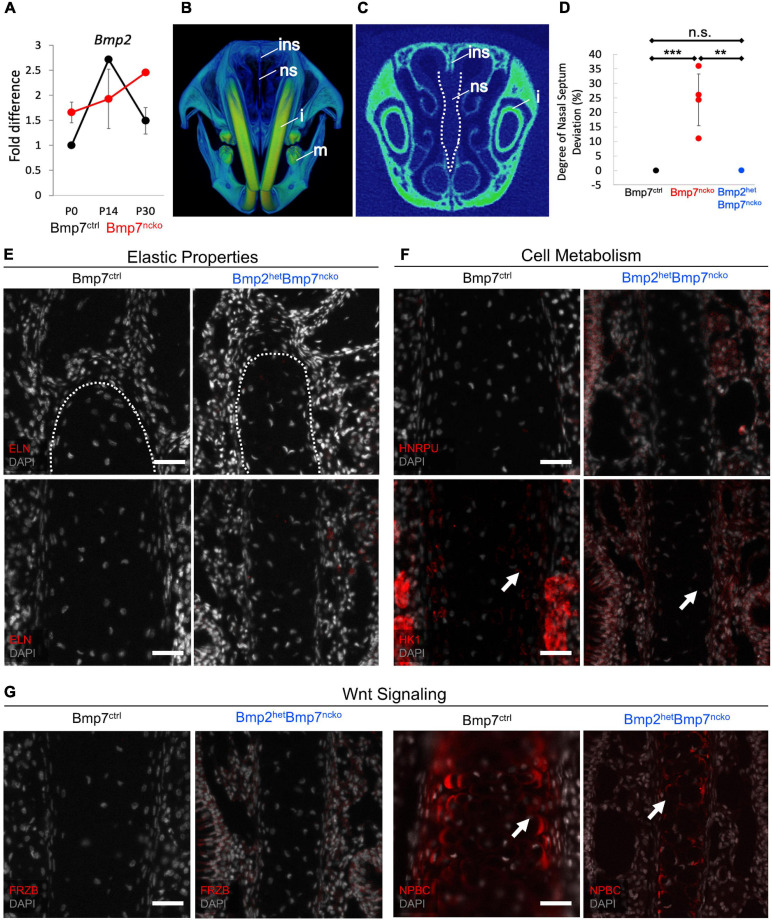
Reduction of BMP2 in Bmp7^*ncko*^ mice rescues the nasal septum deviation. **(A)** qRT-PCR of P0, P14, P30 dissected nasal septum revealed an increase in BMP2 expression at P30 in Bmp7^*ncko*^ mice. **(B)** 3D frontal view of P30 Bmp2^*het*^Bmp7^*ncko*^ mouse demonstrating a straight nasal septum. **(C)** 2D overview of nasal septum (outlined in white). **(D)** Quantification of nasal septum deviation in Bmp2^*het*^Bmp7^*ncko*^ mice revealed no significant difference between Bmp7^*ctrl*^ and Bmp2^*het*^Bmp7^*ncko*^. Note that the data for Bmp7^*ctrl*^ and Bmp7^*ncko*^ is repeated from Figure 1L. **(E)** No expression of Elastin (ELN) was observed in P30 Bmp2^*het*^Bmp7^*ncko*^ mouse. **(F)** Immunofluorescence staining of Heterogeneous nuclear ribonucleoprotein U (HNRPU) in P30 Bmp7^*ctrl*^ and Bmp2^*het*^Bmp7^*ncko*^ mice was comparable, however, staining with Hexokinase (HK1) showed a reduction in expression of HK1 in the Bmp2^*het*^Bmp7^*ncko*^ mouse. **(G)** Expression of FRZB between P30 Bmp7^*ctrl*^ and Bmp2^*het*^Bmp7^*ncko*^ mice was comparable but a slight reduction in Non-Phosphorylated Beta-Catenin (NPBC) was observed in Bmp2^*het*^Bmp7^*ncko*^ mice. However, both Bmp7^*ctrl*^ and Bmp2^*het*^Bmp7^*ncko*^ mice demonstrated cytoplasmic expression of NPBC. Ins, internasal suture; ns, nasal septum; i, incisor; m, mandible. Independent two-tailed *t*-test was performed with significance denoted as *: *P* < 0.05, **: *P* < 0.01, ***: *P* < 0.001. (*n* = 3/genotype/age). Antibody expression demonstrated in red and DAPI stained nuclei in gray for all immunofluorescence images. Arrows denoting positive antibody signal. Scale bar = 50 μm.

While assessing changes to the BMP signaling pathway (Figure 5C), we also tested for changes in BMP2 expression, another BMP involved in cartilage development (Gamer et al., 2018). Indeed, BMP2 was increased at P0 (*p* < 0.01), failed to be induced at P14 but remained significantly increased at P30 (*p* < 0.05) (*n* = 3/age/genotype) (Figure 8A). BMP2 and BMP7 have been shown to exert opposite effects on chondrocyte hypertrophy induction (Caron et al., 2013). To assess if the increase in BMP2 directly contributes to the development of NSD, we genetically reduced BMP2 in BMP7^*ncko*^ mice (subsequently BMP2^*het*^BMP7^*ncko*^). To our surprise, the nasal septum in P30 BMP2^*het*^BMP7^*ncko*^ mice remained straight (Figures 8B,C), the deviation observed in BMP7^*ncko*^ appeared to be completely rescued (*p* < 0.01) (Figure 8D). Some key changes identified in the deviated septum were also fully or partially rescued (Figures 8E–G). ELN was not induced anymore (Figure 8E), HNRPU and HK1 demonstrated a partial reversal (Figure 8F), while FRZB and NPBC were comparable to BMP7^*ctrl*^ mice (Figure 8G). These findings indicate that the net balance of BMP signaling is critical for chondrocyte differentiation and that this balance also affects canonical WNT signaling, a key regulator of energy metabolism, including glycolysis.

**FIGURE 9:**
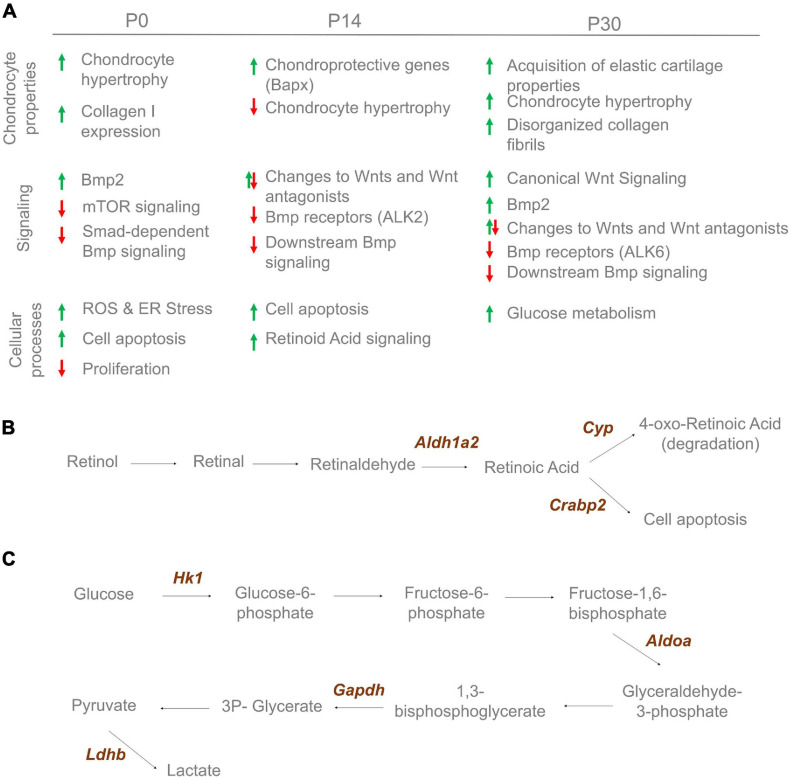
Cartilage changes observed in Bmp7^*ncko*^ mice. **(A)** Altered cartilage properties, signaling and cellular processes observe over-time in Bmp7^*ncko*^ mice. Proteomics analysis revealed proteins involved in retinoic acid signaling at 2 weeks **(B)** and glucose metabolic process at 4 weeks **(C)**.

Together, our data establish an unexpected complex etiology for the development of NSD involving both BMP and WNT signaling. Changes to these pathways are associated with changes to chondrocyte specification, subsequent differentiation involving many cellular pathways including cell metabolism as summarized in Figure 9. Thus, a detailed assessment of extracellular matrix, cell metabolism and signaling properties are required when developing effective treatments and tissue engineering approaches for septoplasties.

## Discussion

This study provides the first detailed characterization of the molecular and cellular etiology of NSD in a well-characterized model of midfacial hypoplasia associated with nasal airway obstruction (Baddam et al., 2021a). We uncovered that the deviated cartilage expressed several markers abundant in elastic cartilage and showed hallmarks commonly associated with osteoarthritic cartilage. This suggests a fundamental change in cartilage properties and highlights the complex etiology of NSD and likely has ramifications for other hyaline cartilage-related pathologies.

Nasal septum deviation is classified based on the type of shape changes (Teixeira et al., 2016; Taghiloo and Halimi, 2019). Some forms of NSD result from uncoordinated growth of the septum and its articulating structures (vomer, ethmoid, nasal bones) resulting frequently in facial asymmetries. While in those cases underlying cartilage defects are unlikely, cartilage defects have been proposed in other situations (Teixeira et al., 2016). Why in some cases the septal hyaline cartilage would catastrophically fail has remained unclear. In the BMP7^*ncko*^ mouse, the initially straight septum gives way to a C-shaped kink, which is additionally associated with a growth defect resulting in midfacial hypoplasia (Baddam et al., 2021a). C or S-shaped deviations are commonly observed in neonates. It is unclear to what degree cartilage growth abnormalities are already pre-existing *in utero* or can be attributed to trauma to the face during birth (Harugop et al., 2012).

Nasal cartilage growth in mice occurs in two phases. Rapid growth between P0-P14 and from P21 onward (Hall and Precious, 2013; Baddam et al., 2021b) is interrupted by a period of limited to no growth in between. A similar bimodal nasal growth occurs also in humans with significant growth occurring between 3–4 years and 11–12 years (Albert et al., 2019). The onset of septum deviation in BMP7^*ncko*^ mice prior to the second growth phase coincides with the increased prevalence of NSD in adolescents (Baddam et al., 2020). While the first phase of septum growth is governed by cell proliferation, growth during the second phase occurs predominantly through cell hypertrophy (Baddam et al., 2021b). BMP7 is expressed in and around the nasal cartilage at all stages, with the lowest expression observed at the P14 time-point. Given this cellular and molecular heterogeneity, we investigated three time-points (P0, P14, P30) to understand if and when changes to the nasal cartilage manifest. Discrete changes were already observed at birth when chondrocytes were still immature (Gómez-Picos and Eames, 2015; Baddam et al., 2021b) suggesting that BMP7 controls aspects of early chondrocyte differentiation, although the forming cartilage is histologically inconspicuous until at least P14. The largest proteome and gene expression differences were observed at P14. Substantial apoptosis was accompanied by reduced protein levels for markers promoting chondrocyte hypertrophy (RUNX2 and IHH) and increased presence of proteins inhibiting hypertrophy (BAPX, FRZB, GREM1) in BMP7^*ncko*^ mice. This picture was changed at P30, where an increase in proteins indicative of chondrocyte hypertrophy (COLX and IHH) was observed. Thus, the earliest changes identified already suggest a change in chondrocyte properties toward an elastic cartilage phenotype. Around P14, when septum growth switches from proliferation to hypertrophic growth, this change leads to catastrophic apoptosis, midfacial hypoplasia, and because of altered chondrocyte properties, septum deviation. Our data is compatible with a transformation of hyaline cartilage to elastic cartilage and a subsequent loss of mechanical resistance. The reduction of collagen fibril network at the deviated side is suggestive of reduced stiffness (Wong et al., 2018). The contralateral increase in collagen fibrils might indicate an attempt to stabilize the septum. Interestingly, both BMP7 and GREM1 (Yu et al., 2017) are expressed at sites of Elastin expression, suggesting that these genes may contribute to chondrocyte remodeling and cartilage specification. Most of the changes were initially observed in the perichondrium rather than mature chondrocytes, the site where cartilage progenitor cells reside (Kaucka et al., 2017). Cell death within the cartilage may activate these chondrocyte progenitor cells to replace dying cells (Seol et al., 2012). These perichondrial cells express MFAP5, another gene found in elastic cartilage (Asnaghi et al., 2020). We propose that these MFAP5 expressing cells in the perichondrium precipitate the proposed switch from hyaline to elastic cartilage. More detailed lineage tracing of perichondrial-derived cells will be required to shed further insights into the mechanism underlying the cartilage switch.

We used quantitative shotgun proteomics to characterize cellular consequences of this change in cartilage properties and understand why chondrocytes might undergo apoptosis. Focusing on cellular metabolism, we found that mTOR, an established suppressor of autophagy (Chen and Long, 2018), was decreased at P14. Several members of the cytochrome P450 (CYP) family (Yoshinari et al., 2004; Mao and Zhang, 2018), which are involved in the maintenance of white adipose tissue, and other proteins involved in retinoic acid signaling and lipid metabolism were increased. BMP7 has an established role in adipocyte lineage determination, promoting brown adipogenesis at the expense of white adipocytes (Schulz and Tseng, 2009). These two adipocyte lineages are characterized by very different cell metabolism, with brown adipocytes specialized in thermogenesis through uncoupling mitochondrial energy production. It is thus possible that BMP7 in nasal chondrocytes similarly controls specification of different cartilage-types.

An increase in autophagy and metabolic changes have been described to delay the progression of OA (Luo et al., 2019). Thus, these two changes might reflect protective mechanisms to delay cartilage degradation in the septum. It is not clear whether all or only a subset of chondrocytes shows these changes, whether these changes occur within the same chondrocyte, and whether these changes are directly associated with the observed cell death. A clue might come from the P30 data where an increase in classical chondrocyte hypertrophy markers (IHH, COLX), as well as an upregulation of proteins involved in glycolysis, was observed. Hollander and Zeng (2019) demonstrated that hypertrophic chondrocytes express higher levels of glyceraldehyde-3-phosphate dehydrogenase (GAPDH) and lactate dehydrogenase (LDH), two proteins upregulated in P30 BMP7^*ncko*^ mice. It is possible that the cells surviving at P14 might be those that switched to glycolysis, and the cells with increased lipid metabolism are destined to die. While further studies are required to resolve these possibilities, our data strongly suggest that BMP7 controls early chondrocyte differentiation along different developmental trajectories (hyaline versus elastic cartilage). Whether these trajectories correspond to truly different cell lineages as described for brown/white adipocytes needs to be shown.

It has been shown that BMP7 is not the only BMP controlling adipocyte differentiation. BMP2 is an inducer of white adipose tissue (Blázquez-Medela et al., 2019). BMP2 and BMP7 belong to different BMP subgroups. Whereas the former is the ortholog of *Drosophila Melanogaster* Decapentaplegic, latter is the ortholog of glass-bottom-boat and belongs to the 60A group (Malik et al., 2020). In BMP7 control septum, BMP2 was dynamically expressed during nasal septum maturation, with peak expression seen at P14, and expression was low at P30. In the BMP7^*ncko*^ mouse, BMP2 induction at P14 was stunted, but expression was substantially increased at P30. The increase in BMP2 at P30 in BMP7-deficient cartilage might suggest an attempt of molecular compensation. If so, the reduction of BMP2 in BMP7^*ncko*^ mice would be expected to lead to more severe NSD. BMP2/BMP7 double neural crest-knockout mice die at birth. We thus created BMP2 heterozygous/BMP7^*ncko*^ mice. The reduction in BMP2 prevented acquisition of elastic markers and partially restored cell metabolism. In primary chondrocytes, BMP2 has been shown to increase glucose metabolism during endochondral ossification (Lee et al., 2018), hypertrophy in chondroprogenitor cells (Caron et al., 2013) and has been associated with cartilage degradation in OA cartilage (Mariani et al., 2014). We thus propose that the balance between BMP2 and BMP7 rather than the individual signals controls chondrocyte metabolism and differentiation. Our findings not only illustrate how metabolic requirements change during chondrocyte maturation, but that metabolic differences themselves appear to be associated with different cartilage properties. Whether altered metabolism drives the change in chondrocyte properties or is a consequence of altered chondrocyte properties remains to be established. Chondrocyte hypertrophy (COLX, IHH) and change to glucose metabolism observed in the deviated septum are hallmarks of knee OA (van der Kraan and van den Berg, 2012). In addition, several other similarities were observed. COL2 and OPN are reduced in both knee OA (Tsolis et al., 2015; Liu et al., 2020) and NSD. COL VI, a pericellular matrix protein upregulated in OA (Alexopoulos et al., 2009) was also increased in NSD. Alterations in the extracellular matrix may affect mechanosensation leading to cellular stress (Tsolis et al., 2015) and might directly contribute to an increase in apoptosis.

WNT signaling controls cell growth, differentiation, motility, and cell metabolism. In cartilage, canonical WNT signaling has been shown to promote chondrocyte hypertrophy (Mariani et al., 2014; Houben et al., 2016; Liu et al., 2016). As BMP and WNT signaling often cross-talk (Choe et al., 2013; Malik et al., 2020), we probed for changes in WNT pathway genes. Loss of BMP7 led to a changed expression of several WNT ligands (increase in WNT6 and WNT7A, decrease in WNT3A), WNT antagonists (increase in FRZB, decrease in DKK1), and overall activation of canonical WNT signaling evidenced by nuclear localization of non-phosphorylated β-catenin (Choe et al., 2013). The reduction in BMP2, in addition to preventing Elastin expression and metabolic changes, also readjusted canonical WNT signaling. Which of these effects is a direct consequence of restored WNT signaling or is the direct result of rebalanced BMP signaling is currently unclear. For instance, septum deviation is associated with increased mechanical strain (Yang et al., 2017), which itself is linked to an increase in canonical WNT signaling (Brunt et al., 2017). However, the rescue of NSD highlights the need to understand how this balanced signaling controls normal nasal and possibly wider midfacial growth. Identifying commonalities and differences between BMP and WNT signaling will be critical to get a better handle on this molecular complexity. Nevertheless, this study clearly establishes the critical involvement of BMP and WNT signaling in the etiology of NSD. The magnitude and timing of alterations in these signaling pathways might determine onset and severity of NSD and its associated symptoms.

In summary, our findings demonstrate that a multifactorial etiology underlies midfacial hypoplasia-associated NSD. At present, it is not clear which of these changes are a direct consequence of lack of BMP7, and which changes develop as consequence. It is also not clear whether cellular changes (e.g., cell metabolism, hypertrophy) or associated tissue level changes (e.g., thinner septum) predispose or lead to septum deviation. The BMP7^*ncko*^ mouse model presents a genetic basis relevant for cartilage development and pathologies pertaining to not just the nasal septum but hyaline cartilage in general. It also points to the possibility that cartilage type is specified at an early developmental stage and that BMP7 might be a contributing factor. Our data further suggest that cartilage from different origins may share similarities but are differently programmed to meet the requirements of the location the cartilage resides in. The similarities observed in NSD to OA validate to some extent the beneficial outcomes observed when nasal cartilage is transplanted in place of articular cartilage to treat OA. Henceforth, the changes described in BMP7^*ncko*^ mouse model pre- and post- establishment of septum deviation could be relevant for better understanding of OA and other cartilage pathologies. Significant advances in understanding endochondral ossification and cartilage transdifferentiation into bone have been made; however, it is still not clear how different types of cartilages are specified from chondrocyte progenitors. Understanding cartilage lineage specification will alleviate the clinical challenge of fibrocartilage being formed despite efforts to regenerate hyaline cartilage to treat OA (Armiento et al., 2019). Additionally, cell-based tissue engineering strategies such as autologous chondrocyte implantation (ACI) (Brittberg et al., 1994; Mumme et al., 2016) or its more recent variant, matrix-assisted chondrocyte implantation (MACI) (Behrens et al., 2006; Knutsen et al., 2007; Zheng et al., 2007), to repair focal articular cartilage lesions will benefit strongly from understanding the cellular processes that simultaneously promote hyaline cartilage and mitigate fibrocartilage formation.

## Data Availability Statement

The proteomic data has been deposited to the ProteomeXchange Consortium via the PRIDE partner repository under accession number PXD024813. For the RNAseq data, Raw sequencing data is publicly available from the NCBI SRA portal under accession number PRJNA622501.

## Ethics Statement

The animal study was reviewed and approved by Health Sciences Animal Care and Use Committee at the University of Alberta (protocol #: AUP1149).

## Author Contributions

PB: designing research studies, conducting the experiments, acquiring data, analyzing data, writing the manuscript, and editing the manuscript. DY: conducting the experiments, acquiring data, analyzing data, writing the manuscript, and editing the manuscript. GD: analyzing data and editing the manuscript. CN: analyzing data and editing the manuscript. FE: conducting the experiments and editing the manuscript. SE: editing the manuscript. JJ: conducting the experiments, providing reagents, acquiring data, analyzing data, and editing the manuscript. AA: designing research studies, analyzing data, and editing the manuscript. AD: analyzing data, providing reagents, editing the manuscript, and funding acquisition. DG: designing research studies, providing reagents, analyzing data, writing the manuscript, editing the manuscript, and funding acquisition. All authors contributed to the article and approved the submitted version.

## Conflict of Interest

The authors declare that the research was conducted in the absence of any commercial or financial relationships that could be construed as a potential conflict of interest.

## Footnotes

1 https://string-db.org
