# Supplementary material for: Nasal Septum Deviation as the Consequence of BMP-Controlled Changes to Cartilage Properties
Source: Front Cell Dev Biol. 2021 Jun 24;9:696545. doi: 10.3389/fcell.2021.696545 (PMC8265824; doi:10.3389/fcell.2021.696545)
Supplement: Supplementary file 2 [file Table_3.pdf]

Supplemental Table 3: List of All primer pairs used for qRT-PCR.

Gene Name	Primer Pair	Gene Name	Primer Pair
36B4	GTG TGT CTG CAG ATC GGG TA<br>CAG ATG GAT CAG CCA GGA AG	COL II	TGGAAAAGACGGTGAGACGG<br>GCTCCAGCTTCACCAGGAAT
COL VI	AAGGCCCCATTGGATTCCC<br>CTCCCTTCCGACCATCCGAT	Osteopontin	AATCTCCTTGCGCCACAGAA<br>GCAGTGACGGTCTCATCAGA
RUNX2	GCTCACGTCGCTCATCTTG<br>ACACCGTGTCAGCAAAGC	COL X	TTCTGCTGCTAATGTTCTTGACC<br>GGGATGAAGTATTGTGTCTTGGG
Indian Hedgehog (IHH)	CTCTTGCCTACAAGCAGTTCA<br>CCGTGTTCTCCTCGTCCTT	CRTAC1	CAGTCACCAACTCAGTCCTGC<br>CACAACGATCTCGAAGTCCCC
Osteocalcin	ATAGCTCGTCACAAGCAGGG<br>TGACAAAGCCTTCATGTCCA	BAPX	CAGGCGTAACGCTGTCATC<br>CAAGGACCTGGAGGAGGAA
Emilin3	GAGGTGAGCAACACATTACAGA<br>TCCACGTATTCCTCTAGGAGC	WNT3A	TCACTGCGAAAGCTACTCCA<br>CACCACCGTCAGCAACAG
WNT6	CTCCTACAGTGTGGTTGTCAGG<br>GCGCATCCATAAAGAGTCTTGA	WNT7A	GGCTTCTCTTCGGTGGTAGC<br>TGAAACTGACACTCGTCCAGG
FRZB	AGCCCGGATGACATAGTTGT<br>AGTACTGGACACTGCAGAGGG	DKK1	GTCAGTGTGGTTCTTCTGGGA<br>CCGGGAACTACTGCAAAAAT
MSX1	TCTCGGCCATTTCTCAGTCG<br>AGCTGAGCTGTGGTGAAAGG	GREM1	CAGCTGTTGGCAGTAGGGTC<br>ACAGCGAAGAACCTGAGGAC
ALK2	CCT TCA CAG TGG TCC TCG TT<br>TGC TAA TGA TGA TGG CTT TCC	ALK6	TCC AGA GCT TCG TAA GAG CA<br>ATT TGG CGC TGA GCT ATG AC
PTBP1	ATAAGAAGGAGAACGCACTTGTG<br>TGATGCTTGGACAGTGTAATGC	RALY	ACTCTCGGGTCTTCATCGGAA<br>GCGCTCATTGGCATACTGGA
EIF5A	CCATGTAAGATCGTCGAGATGTC<br>ATTCCGTTTGATGTTGGGGAC	CHUK	GTCAGGACCGTGTTCTCAAGG<br>GCTTCTTTGATGTTACTGAGGGC
TBK1	GGAGCCGTCCAATGCGTAT<br>GCCGTTCTCTCGGAGATGATTC	USF1	CTGAAACCGAAGAGGGAACAG<br>GTTGGGGTCAGGAAAAGTGG
PRMT1	CTTGGCTAATGGGATGAGCCT<br>GCGTTGGGCTTCTCACTACTT	CHTOP	TAGGGCGTGGAGCTATGGG<br>CCCTCTTCCCCGACCTATCA
GLI1	CCAAGCCAACTTTATGTCAGGG<br>AGCCCGCTTCTTTGTTAATTTGA	CD44	TCGATTTGAATGTAACCTGCCG<br>CAGTCCGGGAGATACTGTAGC
LIPE	CCAGCCTGAGGGCTTACTG<br>CTCCATTGACTGTGACATCTCG	GPD2	GACTCTCTCCGTTTGCTCATTAC<br>GGATGTCAAATTCGGGTGTGT
PRDX1	AGTCCAGGCCTTCCAGTTCACT<br>GGCTTGATGGTATCACTGCCAG	MMP13	CTTCTGGCACACGCTTTTCC<br>CTTCTGGCACACGCTTTTCC
COL I	TGTCCCAACCCCCAAAGAC<br>CCCTCGACTCCTACATCTTCTGA	BMP2	GGAAGACGTCCTCAGCGAAT<br>ACGGCTTCTTCGTGATGGAA

Supplemental Table 4: Specification of antibodies used.

Antibody	Catalogue #	Antibody	Catalogue #
Green Fluorescent Protein (GFP)	Ab6556	Proliferating Cell Nuclear Antigen (PCNA)	sc-56
Cleaved Caspase 3 (CASP3)	Cell Signalling 9664s	Collagen II (COL II)	ab185430
Collagen VI (COL VI)	sc-377143	Osteopontin (OPN)	Ab8448
RUNX2	sc-390351	Collagen X (COL X)	sc-59954
Indian Hedgehog (IHH)	sc-271101	Elastin (ELN)	sc-166453
Decorin (DCN)	ab175404	Heat Shock Protein 47 (HSP47)	sc5293
Hexokinase I (HK1)	sc-46695	Heterogeneous Nuclear Ribonucleoprotein U (HNRPU)	sc-32315
Frizzled Related Protein (FRZB)	sc-514350	Dickkopf-Related Protein 1 (DKK1)	ab61034
Non-Phosphorylated Beta-Catenin (NPBC)	Cell Signalling 19807	Mammalian Target Of Rapamycin (MTOR)	Cell signalling 2971
Phosphorylated-Smad (PSMAD)	Cell Signalling 13820s	Alexa Flour 647 goat anti-mouse IgG2a	A21241
Alexa Flour 647 goat anti-mouse IgG1	A21240	Alexa Flour 647 donkey anti-rabbit IgG (H+L)	A31573
Alexa Flour 647 goat anti-mouse IgG2b	A21242	DAPI (Molecular Probes)	D-1306
